# Supplementary material for: Berberine Reduces Lipid Accumulation in Obesity via Mediating Transcriptional Function of PPARδ
Source: Int J Mol Sci. 2023 Jul 18;24(14):11600. doi: 10.3390/ijms241411600 (PMC10380538; doi:10.3390/ijms241411600)
Supplement: Supplementary file 1 [file ijms-24-11600-s001.zip › ijms-2485402-supplementary.pdf]

Figure S1. (**A**) 3T3L1 cell viability after BBR treatment for 48h. (**B**) 3T3L1 cell viability after GSK treatment for 48h. (**C**) The quantitative data regarding siRNA efficiency in 3T3L1 cells. The data represent the mean ± SEM (N=3-4). ***P < 0.001, **P < 0.01, *P < 0.05.

Figure S2. Preliminary data of BBR on HFD mice. (**A**) Mice weight with BBR treatment. (**B-D**) Plasma levels of total cholesterol (**B**), triglyceride (**C**) and fasting blood glucose (**D**). (**E-H**) Mass of SWAT (**E**), EWAT (**F**), liver (**G**) and BAT (**H**). The data represent the mean ± SEM (N=4). ****P < 0.0001, ***P < 0.001, **P < 0.01, *P < 0.05. ns: no significance.

Figure S3. (**A**) Plasma levels of total cholesterol, triglyceride and glucose during obesity model establishment. (**B**) Images of EWAT (a) and SWAT (b). (**C**) The liver lipid vacuoles area percentage. (**D**) Mice energy intake during BBR treatment. (**E**) mRNA levels of Gal-3 and aP2 with BBR and GSK treatment. The data represent the mean ± SEM (N=4). ****P < 0.0001, ***P < 0.001, **P < 0.01, * P < 0.05, ns: no significance.

Figure S4. (**A**) Predicted PPREs on the concerned gene promoters. (**B-D**) ChIP-PCR data of the promoters of *Pparδ* (**B**), *Pparγ* (**C**), *Cebpα* (**D**) and *Ho-1* (**E**). Red arrow indicated the positive result.

Table S1 Primers for qPCR.

Table S2 Primers for promoter amplification and mutation.

Table S3 Primers for ChIP-PCR.

Figure S1

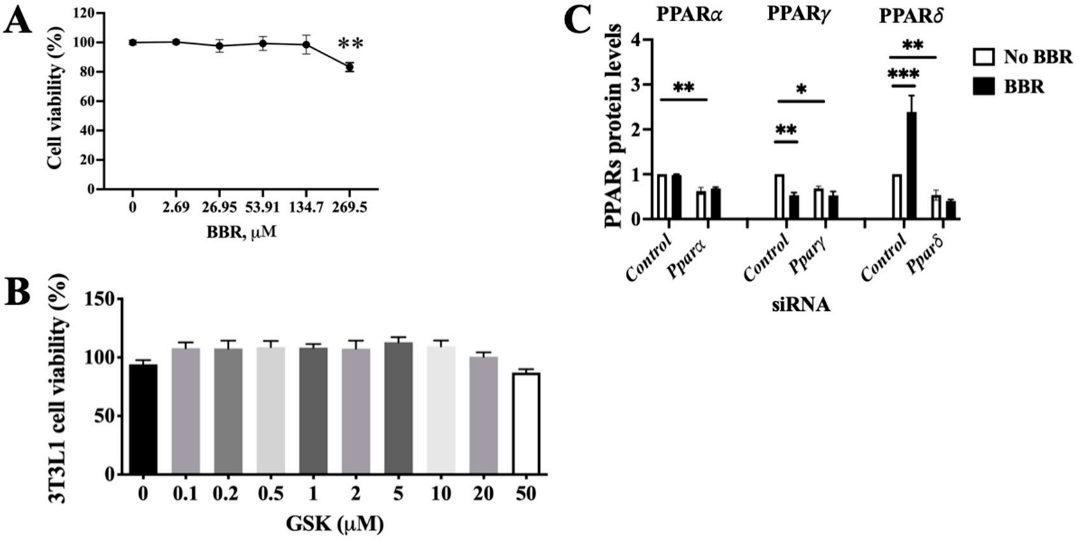
A
Cell viability (%)
120
100
80
60
40
20
0
**
0
2.69
26.95
53.91
134.7
269.5
BBR, μM
B
3T3L1 cell viability (%)
150
100
50
0
0
0.1
0.2
0.5
1
2
5
10
20
50
GSK (μM)
C
PPARα
PPARγ
PPARδ
PPARs protein levels
4
3
2
1
0
**
*
**
**
***
Control
Pparα
Control
Pparγ
Control
Pparδ
siRNA
No BBR
BBR

Figure S2

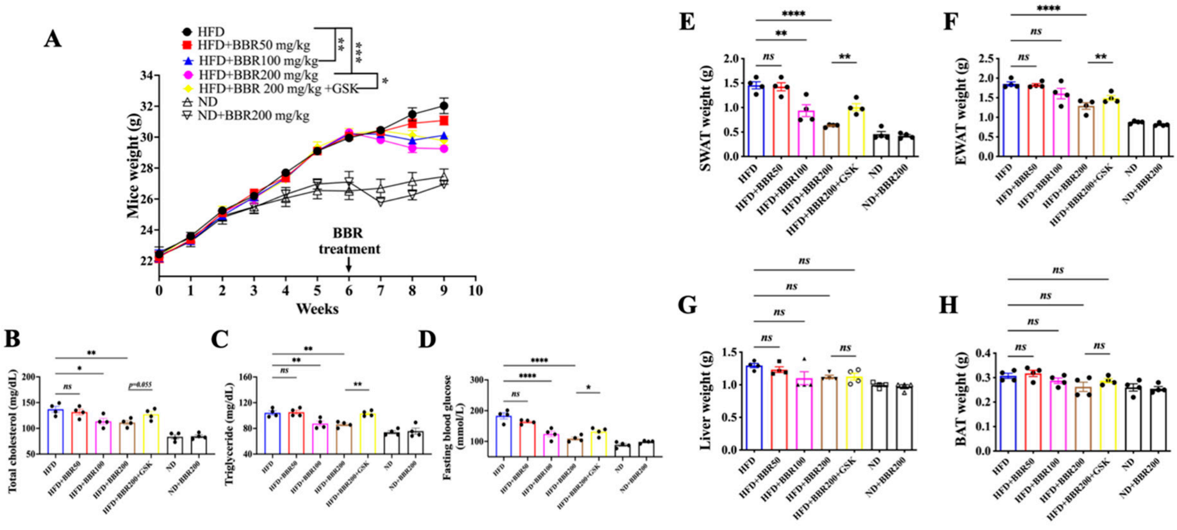

Figure S3

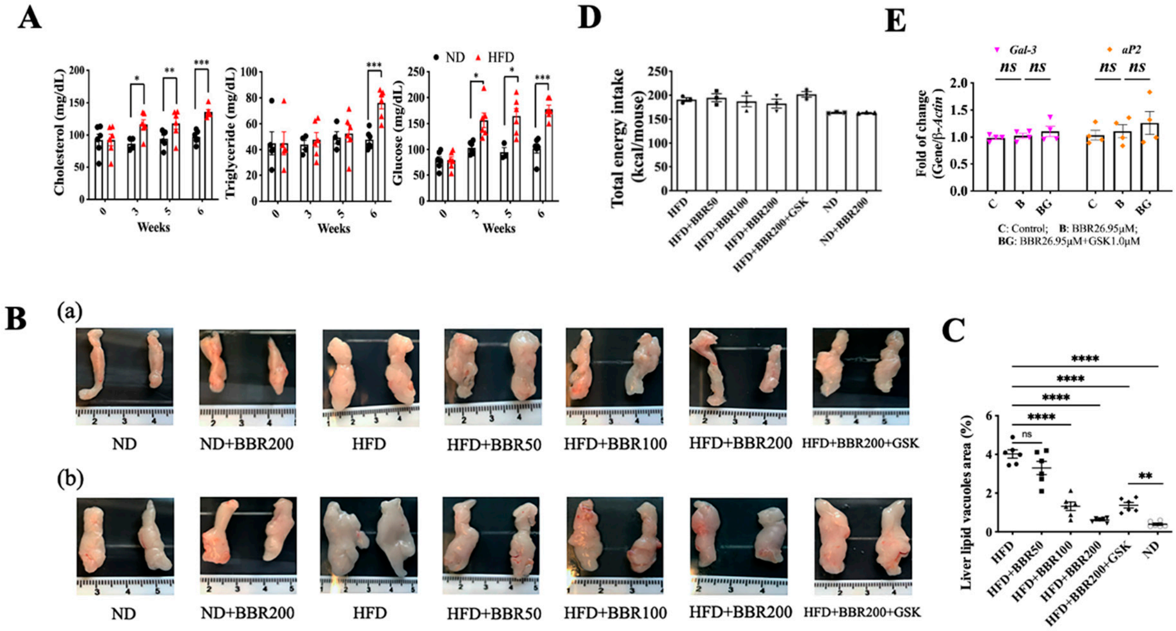
A
D
E
Cholesterol (mg/dL)
Triglyceride (mg/dL)
Glucose (mg/dL)
Weeks
ND
HFD
Total energy intake (kcal/mouse)
HFD
HFD+BBR50
HFD+BBR100
HFD+BBR200
HFD+BBR200+GSK
ND+BBR200
Gal-3
aP2
ns
Fold of change (Gene/β-Actin)
C
B
BG
C: Control; B: BBR26.95μM;
BG: BBR26.95μM+GSK1.0μM
B
(a)
(b)
ND+BBR200
C
Liver lipid vacuoles area (%)
****
**

Figure S4

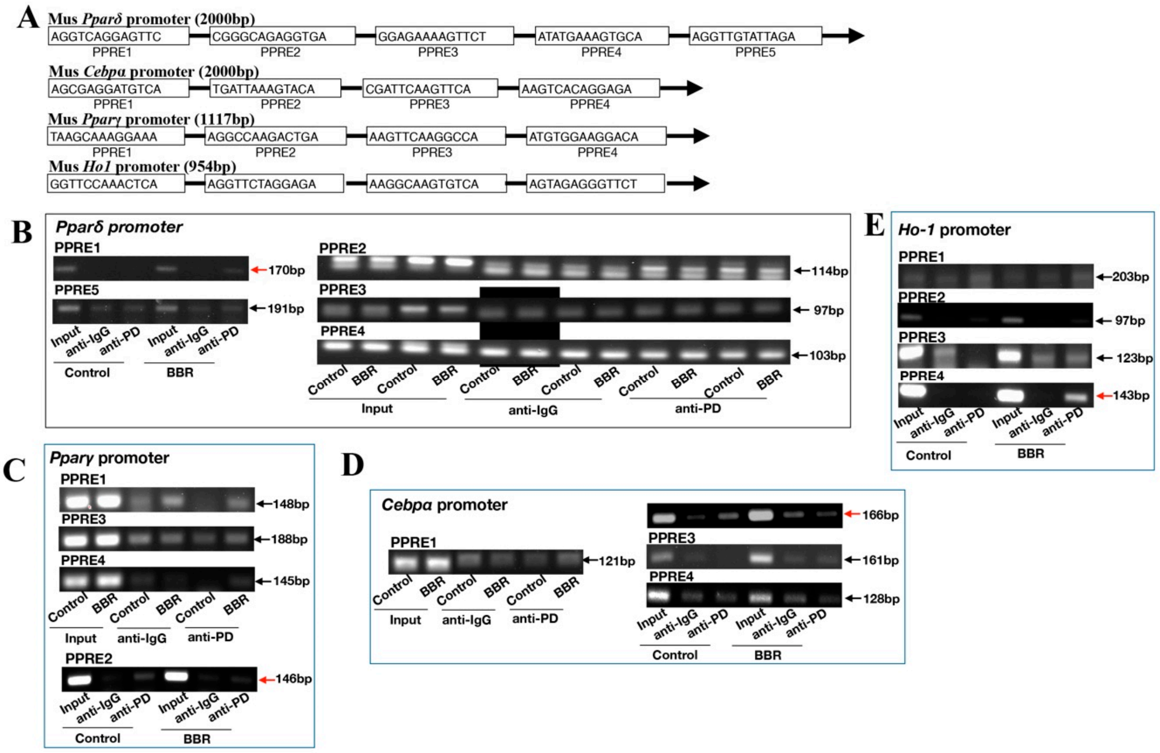
A
Mus Pparδ promoter (2000bp)
AGGTCAGGAGTTC
PPRE1
CGGGCAGAGGTGA
PPRE2
GGAGAAAAGTTCT
PPRE3
ATATGAAAGTGCA
PPRE4
AGGTTGTATTAGA
PPRE5
Mus Cebpα promoter (2000bp)
AGCGAGGATGTCA
PPRE1
TGATTAAAGTACA
PPRE2
CGATTCAAGTTCA
PPRE3
AAGTCACAGGAGA
PPRE4
Mus Pparγ promoter (1117bp)
TAAGCAAAGGAAA
PPRE1
AGGCCAAGACTGA
PPRE2
AAGTTCAAGGCCA
PPRE3
ATGTGGAAGGACA
PPRE4
Mus Ho1 promoter (954bp)
GGTTCCAAACTCA
AGGTTCTAGGAGA
AAGGCAAGTGTCA
AGTAGAGGGTTCT
B
Pparδ promoter
PPRE1
170bp
PPRE5
191bp
Input
anti-IgG
anti-PD
Input
anti-IgG
anti-PD
Control
BBR
PPRE2
114bp
PPRE3
97bp
PPRE4
103bp
Control
BBR
Control
BBR
Control
BBR
Control
BBR
Control
BBR
Control
BBR
Input
anti-IgG
anti-PD
C
Pparγ promoter
PPRE1
148bp
PPRE3
188bp
PPRE4
145bp
Control
BBR
Control
BBR
Control
BBR
Input
anti-IgG
anti-PD
PPRE2
146bp
Input
anti-IgG
anti-PD
Input
anti-IgG
anti-PD
Control
BBR
D
Cebpα promoter
PPRE1
121bp
Control
BBR
Control
BBR
Control
BBR
Input
anti-IgG
anti-PD
166bp
PPRE3
161bp
PPRE4
128bp
Input
anti-IgG
anti-PD
Input
anti-IgG
anti-PD
Control
BBR
E
Ho-1 promoter
PPRE1
203bp
PPRE2
97bp
PPRE3
123bp
PPRE4
143bp
Input
anti-IgG
anti-PD
Input
anti-IgG
anti-PD
Control
BBR

Table S1 Primers for real-time PCR

		Sequence (5'-3')	Accession number
Ppars	*Pparα*	F: TATTCGGCTGAAGCTGGTGTAC	XM_030248424.2
		R: CTGGCATTTGTTCCGGTTCT	
	Pparγ	F: CCACCAACTTCGGAATCAGCT	NM_001308354.2
		R: TTTGTGGATCCGGCAGTTAAGA	
	Pparδ	F: TTGAGCCCAAGTTCGAGTTTG	NM_001411526.1
		R: CGGTCTCCACACAGAATGATG	
Endogenous PPARδ target genes	*Pdk4*	F: CCGCTGTCCATGAAGCA	NM_013743.2
		R: GCAGAAAAGCAAAGGACGTT	
	Ucp2	F: GCTCAGAGCATGCAGGCATCG	NM_001417453.1
		R: CGTGCAATGGTCTTGTAGGCTTCG	
Lipid accumulation related genes	*Cebpα*	F: GCCAAGAAGTCGGTGGACAA	NM_001287514.1
		R: AGTTGCCCATGGCCTTGAC	
	Cebpβ	F: GGGGTTGTTGATGTTTTTGG	NM_001287738.1
		R: CGAAACGGAAAAGGTTCTCA	
	Pparγ	F: CCACCAACTTCGGAATCAGCT	NM_001308354.2
		R: TTTGTGGATCCGGCAGTTAAGA	
	Gata2	F: TGCAACACACCACCCGATACC	NM_001355253.1
		R: CAATTTGCACAACAGGTGCCC	
	Gata3	F: TCTCACTCTCGAGGCAGCATGA	NM_001417048.1
		R: GGTACCATCTCGCCGCCACAG	
	Ho-1	F: AAGCCGAGAATGCTGAGTTCA	NM_010442.2
		R: GCCGTGTAGATATGGTACAAGGA	
	Gal-3	F: CAGTGCTCCTGGAGGCTATC	NM_010705.3
		R: ATTGAAGCGGGGGTTAAAGT	
	aP2	F: CATGGCCAAGCCCAACAT	NM_001409514.1
		R: CGCCCAGTTTGAAGGAAATC	
	β-Actin	F: TGTCCCTGTATGCCT	NM_007393.5
		R: TCACGCACGATTTCCCTC	
		Sequence (5'-3')	Accession number
Ppars	*Pparα*	F: TATTCGGCTGAAGCTGGTGTAC	XM_030248424.2
		R: CTGGCATTTGTTCCGGTTCT	
	Pparγ	F: CCACCAACTTCGGAATCAGCT	NM_001308354.2
		R: TTTGTGGATCCGGCAGTTAAGA	
	Pparδ	F: TTGAGCCCAAGTTCGAGTTTG	NM_001411526.1
		R: CGGTCTCCACACAGAATGATG	
Endogenous PPARδ target genes	*Pdk4*	F: CCGCTGTCCATGAAGCA	NM_013743.2
		R: GCAGAAAAGCAAAGGACGTT	
	Ucp2	F: GCTCAGAGCATGCAGGCATCG	NM_001417453.1
		R: CGTGCAATGGTCTTGTAGGCTTCG	
Adipogenic genes	*Cebpα*	F: GCCAAGAAGTCGGTGGACAA	NM_001287514.1
		R: AGTTGCCCATGGCCTTGAC	
	Pparγ	F: CCACCAACTTCGGAATCAGCT	NM_001308354.2
		R: TTTGTGGATCCGGCAGTTAAGA	
	Gata2	F: TGCAACACACCACCCGATACC	NM_001355253.1
		R: CAATTTGCACAACAGGTGCCC	
	Gata3	F: TCTCACTCTCGAGGCAGCATGA	NM_001417048.1
		R: GGTACCATCTCGCCGCCACAG	
	Ho-1	F: AAGCCGAGAATGCTGAGTTCA	NM_010442.2
		R: GCCGTGTAGATATGGTACAAGGA	

	Gal-3	F: CAGTGCTCCTGGAGGCTATC	NM_010705.3
		R: ATTGAAGCGGGGGTTAAAGT	
	aP2	F: CATGGCCAAGCCCAACAT	NM_001409514.1
		R: CGCCCAGTTTGAAGGAAATC	
	β-Actin	F: TGTCCCTGTATGCCT	NM_007393.5
		R: TCACGCACGATTTCCCTC	

Table S2 Primers for promoter amplification and mutation

Pparδ Wild type	F: CGAATAGGTACCGTTAGGGGAAGGAGTGAGCATGA
	R: GTAAATCCATGGGTCACACGCTCCCAGCC
Pparδ mutant	F: GGAAcGgctGcAGccaAAGACCAGCTCG
	R: TCTTtggCTgCagcCgTTCCTGCCTGC
Cebpα Wild type	F: CGAATAGGTACCCTTATCCCACTTTCTTTG
	R: GTAAATCCATGGGGACCGCTTTTATA
Cebpα mutant	F: ACGCATCCcagACcTcggTtAAATTCTCA
	R: TTTaAccgAgGTctgGGATGCGTCCTTCAGA
Pparɤ Wild type	F: CGCACAGGTACCCTTCTAATTCTAACCCACTGAAGGC
	R: GGAGGGTCCATGGCTCTGGAAATTTATAATTA
Pparɤ mutant	F: CAAtGattAcTGgacAGGATGAGGAGGAAGT
	R: CCTgtcCAgTaatCaTTGGTTCACCATCCAT
Ho-1 Wild type	F: ATACTCGGGTACCATTCCCCTCTGTGGGG
	R: TAGACAAGTCTCGAGGAGCAGCTGCCCGC
Ho-1 mutant	F: GTAGAtTcacGaGTcacGGAAAGGACCCAAAT
	R: GGTCCTTTCCgtgACtCgtgAaTCTACTCAGTGACAGG

Table S3 Primers for ChIP-PCR

Pparδ promoter	
PPRE-1	F: AGCATGAAGCCCTTGTTCCA
	R: AAATGGCCTGGGGAAGTCAG
PPRE-2	F: AAAAAGTGGTGTGGGGT
	R: GTCAGCAGGCACCTCCCT
PPRE-3	F: TCTGGTCCCTGACATCCAGC
	R: CCTGTGCTTATAGGCCTTGTGA
PPRE-4	F: ATATGAAAGTGCACATGCGG
	R: GGGTCATCGCTTTGTCTCCA
PPRE-5	F: TCCCAAGCGCCTCTGAAAAT
	R: AAAGCAAGCCTCACGAAACG
Cebpα promoter	
PPRE-1	F: CCTTCAACCCCAAGTCCCTC
	R: TTGCTTGTTTGGTTCCCCCT
PPRE-2	F: TTTGATTAAAGTACAGGA
	R: AGACCCCCAGTGCAATGGCCT
PPRE-3	F: AGTGCCCCCATGAATGACAG
	R: CTCGCGGAAAAGGACCCTAA
PPRE-4	F: ACTCACCGCCTTGGAAAGTC
	R: CCGCTTTTATAGAGGGTCGGG
Pparɤ promoter	
PPRE-1	F: ACAACCCAGGTGGGCTTTGACA
	R: TGGCCCTTGGTTCACCATCCA
PPRE-2	F: AGGAAGTTTCTCTATAG
	R: TTAACATCTTTATTGAG
PPRE-3	F: CTGACACAAGGGATGGGCT
	R: TTGCTCACCCAGGAGTTTCAA
PPRE-4	F: ATGTGGAAGGACATGAATCT
	R: ATTCTTCTGGTACAAACC
Ho1 promoter	
PPRE-1	F: GGCCTTCTCAGGCACCTATATGC
	R: GAGGCAGAGGCTGGCAGAT
PPRE-2	F: AAGTCATGGTGTGGACC
	R: GGTTGAAGTGTTCTGCCCAG
PPRE-3	F: GGATAAACCATGGAAAAGT

	R: GACCCACACTACCTGAG
PPRE-4	F: CCCTGGGTTTGCCCACCAGC
	R: GCCTCTTGGCAGAATTTGGGTCC
